# How far will they go? Distance and driving times that north American men travel to see a reproductive urologist

**DOI:** 10.1111/and.14551

**Published:** 2022-08-24

**Authors:** Jian Chen, Keith Jarvi, Katherine Lajkosz, James Smith, Susan Lau, Kirk Lo, Ethan Grober, Mary K. Samplaski

**Affiliations:** ^1^ Institute of Urology, University of Southern California Los Angeles California USA; ^2^ Division of Urology, Mount Sinai Hospital University of Toronto Toronto Ontario Canada; ^3^ Department of Urology University of California San Francisco California USA

**Keywords:** access to care, andrology, barriers to care, driving distance, male infertility

## Abstract

Male factor infertility affects about 50% of infertile couples. However, male factor infertility is largely under‐evaluated due to multiple reasons. This study is to determine the time men travel for fertility evaluation, and factors associated with driving longer. Data from the Andrology Research Consortium were analysed. Driving distance and time were calculated by comparing “patient postal code” with “clinic postal code”, then stratified into quartiles. Patients with the longest driving times (> 75th percentile [Q4]) were compared with those having shorter driving times. Logistic regression analysis was used to identify factors associated with longer driving times. Sixteen clinics and 3029 men were included. The median driving distance was 18.1 miles, median driving time was 32 min, and Q4 driving time was 49 min. Factors correlated with having Q4 driving time were age > 30 years, native Indian and Caucasian race, body mass index (BMI) > 30 kg/m^2^, history of miscarriage, children with previous partner, self‐referral, prior vasectomy, and prior marijuana use. On logistic regression, males aged < 30 years were more likely to be in Q4 for driving time versus older males. Blacks and Asians were less likely to travel further than Caucasians. Overweight/obese men, those having children with previous partner, and with prior vasectomy were more likely to be in Q4 travelling time. Factors correlated with longer driving times include younger age, native Indian and Caucasian race, higher BMI, children with prior partner, and prior vasectomy. These may reflect groups that drive long distances for reproductive care. The study provides an opportunity to better access these groups and minimise their barriers to fertility care.

## 1 INTRODUCTION

An estimated 9%–15% of couples struggle with infertility (Agarwal et al., 2015; Boivin et al., 2007). A male factor is responsible in approximately 30% of couples and contributory in another 30%–50% (Agarwal et al., 2015; Boivin et al., 2007). Per the 2020 American Society of Reproductive Medicine guidelines and 2021 European Association of Urology Guidelines on Male Sexual and Reproductive Health, fertility evaluation is indicated after 12 months of unprotected intercourse, or sooner if risk factors or concerns are present. Both partners should undergo concurrent assessment, and males with abnormal semen parameters or presumed male infertility should be evaluated by a male reproductive expert for complete history and physical examination. “Just as all infertile women are treated by those with specialised gynecologic training and expertise, all infertile men be evaluated by specialists in male reproduction” (Schlegel et al., 2021).

However, male factor evaluation by a reproductive urologist (RU) does not happen universally. Data from the National Survey of Family Growth (NSFG) found that amongst couples seeking infertility counselling, 18%–27% of males were not evaluated (Eisenberg et al., 2013). Likewise, only 9.4% of male partners in couples attempting pregnancy had accessed any infertility‐related service (Chandra et al., 2014). Longer infertility duration and white race have been associated with increased odds of male evaluation (Eisenberg et al., 2013).

The possible barriers to RU evaluation are multifactorial, and may include insurance/financial related factors, patient education, cultural and religious beliefs, geography, and RU availability (Mehta et al., 2016). Additionally, lack of patient and provider awareness of male infertility factors or adherence to guidelines, and practise patterns that favour early in vitro fertilisation (IVF), may also limit RU referrals (Cheng & Tanrikut, 2020; Mehta et al., 2016). Often the female partner initiates the fertility evaluation, and reproductive endocrinology and infertility gynaecologists (REIs) have been identified as the “gatekeepers” for male fertility evaluations (Samplaski et al., 2019).

The geographic distance and time that a patient must travel to a RU is of key importance. Whilst telemedicine offers new options for accessing care, for male infertility a physical exam is paramount. Men need to physically access an RU for an exam in order to yield these benefits. One study analysed the RU distribution in relation to the male population aged 20–49 years and found disparities, with large areas being under‐ or overserved (Leung et al., 2016). Similarly, a 2016 study showed that (at publication), 13 states had no male fertility specialists, and many fertility centres did not have a RU within a 60‐min radius (Mehta et al., 2016).

Geographic distance has been shown to impact disease screening, treatment, and survival from urologic cancers (Holmes et al., 2012; Yao et al., 2015). Patients living further from healthcare facilities have worse health outcomes, including survival rates, length of hospital stay and nonattendance at follow‐up visits for urologic care (Leiser et al., 2020). Similarly, lung cancer patients living in rural areas are less likely to attain histologic diagnosis, or receive any treatment, including chemotherapy or surgery (Cabler et al., 2010). Nationally, patients with prostate, kidney and bladder cancers living in areas lacking a urologist have higher cancer‐specific mortalities (Odisho et al., 2010).

Given the importance of male fertility evaluation, and the relative inconsistency of this occurring, as well as the variation in RU distribution, we aimed to understand the effect of travel time on male fertility evaluation. In this study, we sought to determine the geographical distance and driving time that North American men travel to see a RU for fertility consultation, and correlate these with their reproductive care. Also, we seek to identify patient types at greatest risk of exclusion from RU services.

## 2 MATERIALS AND METHODS

Data from the Andrology Research Consortium (ARC) was used. The ARC was established in 2013 by the Society for the Study of Male Reproduction, with the aim to obtain standardised data on the demographics, clinical characteristics, and fertility histories and therapies of North American men undergoing fertility evaluation. A single‐page form was completed by patients at face‐to‐face RU clinic visits, containing fertility related questions. Forms were uploaded to a centralised repository at the University of Toronto. Unanswered questions were left as blank. Results were collated and analysed using descriptive statistics.

Centralised institutional review board (IRB) approval was obtained for centres without a local IRB, and each centre with a local IRB obtained approval for this study.

The field for “patient zip/postal code” was compared with “clinic postal/zip code”. Driving distance and time was calculated using Google Maps for 3 time points: Monday at 8 am, Wednesday at 12 pm, and Friday at 4 pm, of a typical week, in order to account for a variety of appointment times and travel conditions. These driving times were not found to be statistically different, and the Monday 8 am value was used for analyses.

Travel time was stratified into quartiles: Q1 (0%–24%), Q2 (25%–49%), Q3 (50%–74%) and Q4 (75%–100%). Due to the positive skew distribution of the travel time for our cohort, and a relatively small, 11 min, difference between the 25th percentile (21 min) and 50th percentile (32 min), we decided to use the 75th percentile (49 min) as the cut‐off value for comparison.

For each factor of interest, the proportion of patients with driving times above the 75th percentile (Q4) travel time (> 49 min) and below the 75th percentile (Q1–Q3) travel time (< 49 min) were compared using fisher's exact tests. Stepwise univariable and multivariable logistic regression models were constructed to determine factors correlating with patient driving time > 49 min. We first performed univariable analysis to identify the factors associated with longer driving time (> 49 min). We then selected those factors to perform multivariable analysis.

## 3 RESULTS

Sixteen ARC centres and 3029 male patients were included. The median age was 36 years (11–86 years), and body mass index (BMI) was 27.5 kg/m^2^ (13.3–62.9 kg/m^2^). The median driving distance for RU consultation was 18.1 miles (0.2–2623.1 miles), median travelling time was 32 min and 75th percentile driving time was 49 min (2–2580 min). The median driving time of Q1–Q3 was 26 min (IQR 18–35 min). The median driving time of Q4 was 83 min (IQR 60–140 min).

Comparing driving distance and time between the three possible times (Monday 8 am, Wednesday 12 pm and Friday 4 pm), the driving distance and time correlation was nearly perfect with a Spearman correlation coefficient of 0.97 and 0.99 for all 3 days respectively. Therefore, the Monday 8 am driving time was used for analyses.

Factors associated with having a higher proportion of men in the Q4 travel group are seen in Table 1. The youngest men, < 30 years of age, had a higher proportion of men in the Q4 travel time group versus men in other age groups (30–39, 40–49, 50–59 years) (35.3% vs. 23.2%, 24.0%, 20.8%, respectively, *p* < 0.001). Native Indians and Caucasians had a higher proportion of men in the Q4 travel time group than Blacks or Asians (46.4% and 32.5% vs. 11.4% and 13.1%, respectively, *p* < 0.001). Overweight and obese patients with BMI > 30 kg/m^2^ had a higher proportion of men in the Q4 travel time group versus other BMI categories (30.2% vs. 19.4%–24.3%, *p* < 0.001). Couples with history of miscarriage also had a higher proportion of men in the Q4 travel time group (36.7% vs. 25.4%, *p* = 0.04). Other factors associated with having a higher proportion of men in the Q4 travel time group were: men having children with a previous partner (46.1% vs. 22.1%, *p* < 0.001), self‐referred (26.6% vs. 16.2–23.2%, *p* = 0.02), history of vasectomy (49.5% vs. 22.5%, *p* < 0.001), and history of marijuana use (28.7% vs. 23.4%, *p* = 0.011).

**TABLE 1:** Demographics, clinical characteristics, and fertility histories of males driving the longest for RU consultation, above the 75th percentile (Q4) travelling time (> 49 min)

Clinical variable		*n* (%)	*p* value
Age (years)^a^	< 30	106 (35.3)	< 0.001
	30–39	409 (23.2)	
	40–49	186 (24.0)	
	50–59	32 (20.8)	
	≥ 60	7 (24.1)	
Race^b^	Native Indian	13 (46.4)	< 0.001
	Asian	77 (13.1)	
	Black	22 (11.4)	
	Caucasian	502 (32.5)	
	Other	111 (18.1)	
BMI (kg/m^2^)^c^	Underweight/normal (< 25.0)	152 (19.4)	< 0.001
	Overweight (25.0–29.9)	277 (24.3)	
	Obese (≥ 30)	295 (30.2)	
Number of children	0	571 (25.0)	
	≥ 1	52 (24.2)	0.869
Number of pregnancies	0	531 (24.3)	
	≥ 1	104 (28.1)	0.119
Number of pregnancy losses	0	59 (25.4)	
	≥ 1	40 (36.7)	0.04
Children with current partner	No	696 (24.5)	
	Yes	45 (23.8)	0.862
Children with previous partner	No	604 (22.1)	< 0.001
	Yes	137 (46.1)	
Referral source^d^	Self‐referred	33 (26.6)	0.019
	PCP	33 (16.8)	
	REI	225 (23.2)	
	Other	32 (16.2)	
	No	634 (22.5)	
History of vasectomy	Yes	107 (49.5)	< 0.001
	No	610 (25.5)	
Partner with prior intrauterine insemination	Yes	66 (20.6)	0.055
	No	650 (24.9)	
Partner with prior in vitro fertilisation	Yes	61 (26.4)	0.635
	No	509 (24.2)	
History of smoking	Yes	128 (27.5)	0.138
	No	275 (23.7)	
History of alcohol use	Yes	445 (24.9)	0.483
	No	571 (23.4)	
History of marijuana use	Yes	162 (28.7)	0.011
	No	710 (24.5)	
History of cocaine use	Yes	15 (29.4)	0.414
	No	735 (24.5)	

^a^ Patients < 30 years old had significant higher Q4 travelling time versus patients in other age groups (30–39, 40–49, 50–59 years old) (35.3% vs. 23.2%, 24.0%, 20.8%, respectively, *p* < 0.001).

^b^ Native Indians had higher Q4 travelling time than African Americans or Asians (46.4% vs. 11.4%, 13.1%, respectively, *p* < 0.001). Caucasians had higher Q4 travelling time than African Americans or Asians (32.5% vs. 11.4%, 13.1%, respectively, *p* < 0.001).

^c^ Patients with BMI > 30 kg/m^2^ (obese) had higher Q4 travelling time than other BMI categories (underweight/normal, overweight) (30.2% vs. 19.8%, 24.3%, respectively, *p* < 0.001).

^d^ Significant difference was detected amongst all categorical groups of referral sources, but no significant difference was detected at pairwise comparison.

On univariate analysis, factors associated with higher proportion males with Q4 travelling time were age (*p* < 0.001, however, no difference detected in pairwise comparison amongst different age groups), overweight status (BMI 25.0–29.9 kg/m^2^) (OR = 1.31 [1.04–1.63], *p* = 0.002), obese (BMI ≥30 kg/m^2^) (OR = 1.76 [1.4–2.2], *p* < 0.001), children with previous partner (OR = 3.02 [2.36–3.86], *p* < 0.001), referral source (*p* = 0.026), history of vasectomy (OR = 3.37 [2.55–4.47], *p* < 0.001), and history of marijuana use (OR = 1.32 [1.07–1.61], *p* = 0.009). Blacks (OR = 0.27 [0.17–0.42], *p* < 0.001) and Asians (OR = 0.35 [0.24–0.41], *p* < 0.001) had less males in the Q4 travelling time group.

**FIGURE 1:**
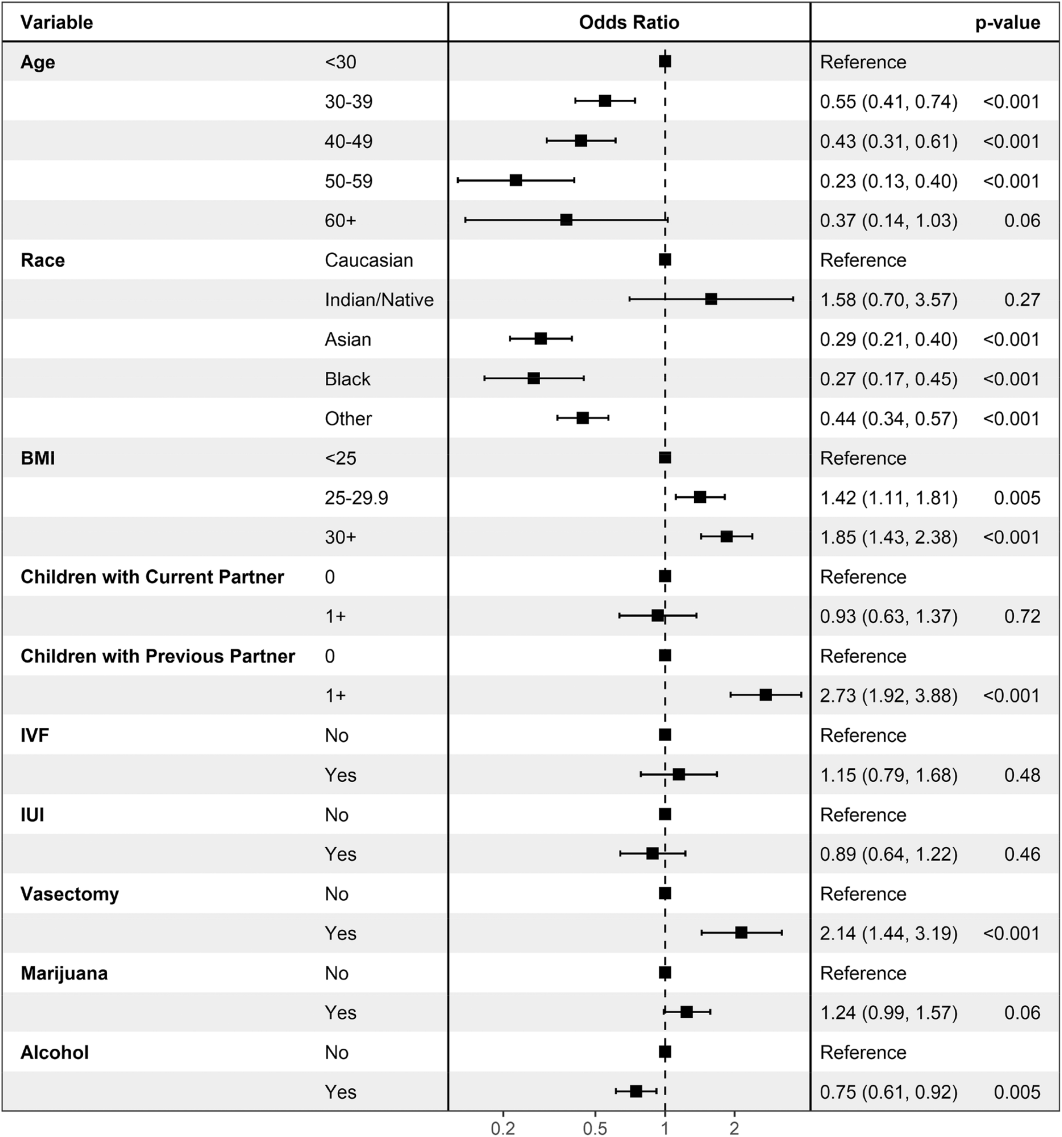
Forest plot of multivariable analysis, showing factors correlated with males driving the longest for RU consultation, above the 75th percentile (Q4) travelling time (> 49 min)

After adjusting for the above factors and other clinically significant covariates (children with current partner, prior IVF), the following variables remained statistically significant for being more likely to have more patients in the Q4 travel time group with *p* < 0.001 (Figure 1): Weight status, specifically overweight (BMI 25–29.9 kg/m^2^) and obese (BMI > 30 kg/m^2^) patients (OR = 1.42 (95% CI: 1.11–1.81), 1.85 (95% CI: 1.43–2.38), respectively, *p* ≤ 0.005); History of children with previous partner (OR = 2.73 [95% CI: 1.92–3.88], *p* < 0.001); History of vasectomy (OR = 2.14 [95% CI: 1.44–3.19], p < 0.001). Factors predictive of less being less likely to have patients in the Q4 travel time group were: Patients aged 30–39, 40–49, and 50–59 years (OR = 0.55 (95% CI: 0.41–0.74), 0.43 (95% CI: 0.31–0.61), 0.23 (95% CI: 0.13–0.40), respectively, *p* < 0.001); Blacks and Asians (OR = 0.29 (95% CI: 0.21–0.40), 0.27 (95% CI: 0.17–0.45), respectively, *p* < 0.001). Men with alcohol use were also shown to be associated with less Q4 travelling time (OR = 0.75 [95% CI: 0.61–0.92], *p* = 0.005). History of pregnancy loss and referral source were not included in the multivariable analysis due to high rates of missing data.

## 4 DISCUSSION

The reasons for a male factor evaluation are multifactorial, including both the immediate fertility goals and also long‐term male health. RU consultation may detect reversible pathologies to improve natural pregnancy outcomes (Farber et al., 2019) or optimise sperm quality for couples using assisted reproductive technologies (Cayan et al., 2002). RU evaluation may also identify underlying medical and genetic pathologies that may affect the patient's long‐term health or that of his offspring (Krausz et al., 2015).

Whilst access to male fertility care is a multidimensional issue, one of the most important factors to consider is physical distance. Data from the 2000 US Census Bureau found wide variation in geographic access to a RU, defined as 60‐min travel radius. Some areas were underserved, and others were overserved, and 13 states had no RU whatsoever (Nangia et al., 2010a; Nangia et al., 2010b).

The COVID‐19 pandemic has changed health systems worldwide, notably for the explosion in telemedicine. A growing body of literature has shown the successful applicability of this technology for urology, and specifically for andrology (Shiff et al., 2021). Whilst majority men undergoing fertility evaluation do need one clinic visit for a face‐to‐face physical exam, many visits may be done via telemedicine. For example, a male patient with need for sperm extraction due to vasectomy could potentially be effectively evaluated via telemedicine, with the aid of scrotal ultrasound images in addition to other relevant laboratory results. This would be sufficient prior to scheduling surgery. Another possible example is telemedical consultation of a male with Klinefelter syndrome established on laboratory testing and scrotal ultrasound imaging prior to scheduling testicular micro dissection. Whilst the growing use and acceptance of telemedicine, patients will have greater access to a broader range of physicians. Whether this translates into more male fertility patients being evaluated by a RU remains to be seen. Interestingly, recent data showed that patients using telemedicine for urology were more likely to live further from the clinic, and that andrology was one of the most common urologic specialties to use telemedicine (Andino et al., 2020).

We found that younger men, < 30 years, were more likely to drive further for RU consultation. There is currently no other published data looking at age and driving distance for medical care. We do know that reproductive aged males are 2 to 2.5 times less likely than women to visit a doctor (Eisenberg et al., 2013). Concurrently, Berger, et al. found that younger patients are three times more likely to utilise internet resources to select a urologist (Berger et al., 2020). The prolonged driving distance for younger patient in our study may reflect an Internet search a willingness to travel greater distances with the assistance of map‐based app technologies. The correlations may also reflect that RU tend to locate in more affluent communities and younger men are less likely to afford to live in the wealthier communities than older men and therefore may need to travel longer distances.

Males in the Q4 driving group were more likely to be self‐referred, versus referred by REI or primary care physician. Prior ARC data has shown that REIs are the gatekeepers for male infertility care, and that 59.7% of males seen by a RU were referred by an REI (Samplaski et al., 2019). In addition, a survey of infertility treatment centre websites found that urology referral for male evaluation was mentioned by < 25% of websites (Leung et al., 2016). It would stand to reason that REIs and primary care physicians would refer males to RUs that are geographically in close proximity. We found that self‐referred males were more likely to drive further, which may reflect Internet searching a RU and potentially driving longer distances. This may also explain our finding that men with a prior vasectomy drive longer distances to see a RU. Since both vasectomy reversal and sperm retrievals are often cash procedures, men may be motivated to work with a RU that they believe has the highest odds of success, and if needed drive a longer distance for that care. Also, not all urologists are trained to do vasectomy reversals, which might also be a reason patients have to go far distances for the procedure.

We found that certain racial groups, Native Indian and Caucasian, were more likely to travel greater distances, versus Blacks or Asians. American Indian and Alaska Native women have been found to have more limited geographic access to critical care obstetrics care compared with other racial groups (Kroelinger et al., 2020). Finally, there is some data to show that Black patients are more likely to report cost as a deterrent to seeking health care versus Caucasian patients (Chaudhry et al., 2011). Given that fertility care is not covered by many insurances, some racial groups may not have the transportation resources or employment flexibility to drive great distances for care that may be costly. Insurance and hospital referral networks may also play into the geographic access to RU care.

Interpersonal relationship factors, or social misnomers may also play a role in some of our findings. There is common misperception that infertility is primarily a “woman's problem”. We found that males with a history of female partner with miscarriage were more likely to drive greater distances. The pain of miscarriage, and desire to prevent future miscarriages, may motivate some males to get a RU evaluation. There is data to show that both male and female depressive scores correlate with the number of prior infertility treatment cycles (Yoldemir et al., 2020), which may motivate some couples to have both partners evaluated. We also found that men with secondary infertility and a child with a different partner were more likely to drive a greater distance for RU consultation. This may reflect the desire to achieve a live birth with their current partner and meet the standard that was set with the first partner.

Males who were overweight or obese were also more likely to drive further for RU consultation. This may reflect a male desire to optimise sperm quality to contribute to a pregnancy. A growing body of evidence shows that male obesity may lead to subfertility, from endocrinopathies, increased testicular temperature, decreased sperm quality and vasculogenic erectile dysfunction (Cabler et al., 2010). Overweight males may seek RU care in order to optimise their fertility, in light of their overweight status.

Our study has limitations. Patients working with RUs not represented in the ARC data may have different driving times and distances. We have unproportioned cohort distribution and make the analysis difficult and biassed. We used Google maps to formulate driving distance and time, although individual driving distances and times may vary. Patients using public or air transportation were not accounted for in our analysis. Female partner age is also a potential influencing factor for male patient to seek fertility evaluation, which is not investigated in this study. Finally, the causality for understanding of why these groups emerged as correlating with driving greater distances, is not on our ARC questionnaire.

## 5 CONCLUSIONS

Younger males, having greater BMI, children with a prior partner, and prior vasectomy tended to travel further for RU evaluation. Blacks and Asians were associated with less driving time. Whilst the cause for this disparity is unknown, the longer drive times may result in lost wages or possibly reflect groups that do not drive long distances for reproductive care. This data provides an opportunity for us to better reach these patients, minimise barriers and provide more options to reproductive care for them, especially in the new era of telemedicine.

## AUTHOR CONTRIBUTIONS

**Jian Chen**: Manuscript drafting. **Keith Jarvi**: Study design. **Katherine Lajkosz**: Statistic analysis. **James Smith**: Study design. **Susan Lau**: Data collection. **Kirk Lo**: Data collection. **Ethan Grober**: Data collection. **Mary K. Samplaski**: Study design.

## FUNDING INFORMATION

This study is funded by Society Study Male Reproduction.

## CONFLICT OF INTEREST

The authors declare that there is no conflict of interest.

## Data Availability

The data that support the findings of this study are available on request from the corresponding author. The data are not publicly available due to privacy or ethical restrictions.
